# Associations between chronotype, physical activity and cognition in a free-living setting

**DOI:** 10.1007/s00221-026-07254-5

**Published:** 2026-02-27

**Authors:** Evie Holt, Benjamin Tari, Demi Ojo, Zaina Alavi, Evelyn Watson, Sarah Keating Bartlett, Flaminia Ronca

**Affiliations:** 1 Faculty of Health and Life Science, University of Exeter, Exeter, UK; 2 Institute of Sport, Exercise and Health, Division of Surgery and Interventional Science, University College London, London, UK; 3 Division of Surgery and Interventional Science, University College London, First Floor, 170 Tottenham Court Road, London, W1T 7HA UK

**Keywords:** Accelerometery, Working memory, Executive function, Control, Attention

## Abstract

Chronotype is one’s inherent preferred pattern of sleep and activity within a 24 hour period. Late chronotypes in particular are prevalent among university students and have been associated with differences in physical activity and cognitive performance compared to earlier chronotypes. While these effects have been studied in isolation and in controlled settings, the interaction between chronotype, cognition and physical activity in free-living settings has not yet been studied. The aim of this study was to investigate associations between self-reported chronotype, objective physical activity patterns, and cognitive performance in a naturalistic setting. Seventy-five young adults (age = 25 ± 7 years; 57% female) completed the Composite Morningness Questionnaire to classify chronotype, before wearing accelerometers for seven days. During this time participants completed executive function tasks at the start and end of the day, without modifying their regular sleep-wake hours. Early chronotypes were more physically active compared to late chronotypes, had an earlier time of peak activity, and exhibited shorter reaction times and fewer errors compared to late chronotypes, particularly in the morning. In fully adjusted models, end-of-day testing as well as being active in the hour prior to testing were associated with better cognitive performance. These results suggest that in free living settings, early chronotypes are associated with both better executive function and higher activity levels in the morning, but that activity levels can improve cognitive performance regardless of time of day. Further research could consider whether physical activity could be leveraged to support morning cognitive function in real-world contexts.

**Supplementary Information:**

The online version contains supplementary material available at 10.1007/s00221-026-07254-5.

## Introduction

Individuals can be categorised by chronotype, reflecting their inherent patterns of sleep and activity. On one end of the spectrum are those with early chronotypes, who go to sleep and rise early in the evening and morning, respectively. On the opposite end, late-type chronotypes sleep and rise late in the evening and morning (Evansová et al. 2022). Most of the population falls somewhere in-between and can be characterised as having intermediate, or neutral, chronotypes (Liu et al. 2020). Chronotype is controlled by both biological and environmental factors (Evansová et al. 2022; Roenneberg et al. 2003, 2004), dictating an intrinsic circadian rhythm of physiological processes with a cycle length of approximately 24 h. Associated with this rhythmicity are differences in cognitive and physical performance over the course of the day (Wiłkość-Dębczyńska and Liberacka-Dwojak 2023; Vitale and Weydahl 2017).

This is salient considering the societal expectation for individuals to perform well, both cognitively and physically, at times of day which may be at odds with their periods of chronotype-driven peak performance, specifically early waking hours. Indeed, working hours in Western culture generally consist of an 8-hour day, 09:00 to 17:00, Monday through Friday. This is similar to typical University schedules, and the school day is often even more shifted towards the morning (e.g. 09:00 to 15:00). This mismatch between chronotype and schedule may be greatest in young adults, a period when prevalence of late chronotype has been shown to peak (Fischer et al. 2017; Roenneberg et al. 2004). This may result in social jetlag, a phenomenon whereby there is misalignment of biological and social time (Wittmann et al. 2006) which has recently become a major public health concern (Caliandro et al. 2021).

Supporting this, a body of literature has identified a relationship between chronotype and cognition (Wiłkość-Dębczyńska and Liberacka-Dwojak 2023). Specifically, there is evidence of a synchrony effect wherein an individual’s chronotype aligns with a time of day where cognitive performance is optimal. Late chronotypes generally show worse cognitive performance than early chronotypes in the morning. A synchrony effect has also been shown for academic performance, with several studies showing better academic outcomes for early and late chronotypes following morning and afternoon schooling respectively (Goldin et al. 2020; Rodríguez Ferrante et al. 2023). More recently, a systematic review identified the synchrony effect for cognition in only approximately half of the studies considered, predominantly when assessing attention, inhibition, and memory (Chauhan et al. 2025). This aligns with the conclusions of Wiłkość-Dębczyńska and Liberacka-Dwojak that further research is needed into how chronotype impacts human brain physiology and cognition.

In parallel, there is evidence suggesting an association between later chronotype and adverse lifestyle habits. A systematic review by Sempere-Rubio and colleagues (2022) demonstrated that late chronotype was associated with reduced duration, frequency, and intensity of physical activity, and an increased tendency towards sedentary behaviour, compared to individuals with early chronotypes. Of note, Sempere-Rubio and colleagues highlighted inconsistent results when looking at young adults specifically, and called for further research in this population. This echoes another review from the subsequent year (Vidueira et al. 2023) which found very heterogeneous outcomes concerning the association between physical activity and chronotype and called for further research in this area.

In summary, a body of literature has associated later chronotype, most prevalent at the age when university attendance generally occurs, with lower physical activity and poorer cognitive performance, in particular early in the day. This is interesting considering the known benefits of physical activity for cognition (Erickson et al. 2019). There is a dearth of research bringing together these fields to investigate the associations between chronotype, physical activity and cognition in free-living contexts. One study did so in older adults, however cognition was only assessed at one timepoint and no analyses included both physical activity and cognition (Hicks et al. 2023). A more recent study in young adults evaluated the interplay among chronotype, physical activity and cognition, however their population consisted entirely of late-type individuals and did not include any end-of-day cognitive testing (Marchesano et al. 2025). Therefore, this observational naturalistic study investigated whether chronotype is associated with physical activity behaviours and cognitive performance at the start and end of the day in young adults, and whether these interact. It was hypothesized that later chronotypes would be less physically active and have poorer cognition at the start of the day compared to earlier chronotypes, with cognition improving throughout the day and following bouts of physical activity.

## Methods

### Participants

Young adults (aged 18–30) were recruited via snowball sampling from a cohort of university students and early career researchers (PhD students, early postdoctoral researchers) by word of mouth and email communication. Participants were excluded if they reported having a fixed working schedule, known sleep disorder, neurological disorder or neurodivergence. University students and early career researchers included in the sample had varied schedules, with mostly mid-day or afternoon lectures and occasional 9:00 or 10:00am lectures. The early career researchers and members of the general public included in the study all had control over their schedule and were not tied to specific working hours. A power calculation to detect an effect size of 0.2 (common in cognitive neuroscience behavioural data) between five chronotypes groups with six measurements yielded a required sample of 70 participants. All participants provided informed written consent prior to taking part in the study, which was approved by the University’s Research Ethics Committee (approval number 13985/005) in accordance with the most recent iteration of the Declaration of Helsinki.

## Study design

Data was collected between December 2023 and April 2024 in London. Participants were invited to visit the lab on one occasion where they were familiarised with study procedures. Participants then completed a demographic questionnaire along with the Composite Morningness Questionnaire (Smith et al. 1989) via the Gorilla Experiment Builder (www.gorilla.sc; Anwyl-Irvine et al. 2020). After the lab visit, participants were provided with an accelerometer to wear consecutively for 7 days, and were asked to complete a brief questionnaire and cognitive test on the Gorilla platform from their mobile phones every other day, as close to 09:00 and 21:00 h as possible, without modifying their routine or waking hours. Reminders were sent to participants prior to their required log times via text message. Prior to cognitive testing, participants were asked if they had consumed any caffeinated food or beverage. To avoid any doubt, they were provided with examples (coffee, energy drinks, tea, dark chocolate) and directed to an online calculator which estimated how many milligrams of caffeine they had consumed on the stated beverage or food. If the amount of caffeine was equal or superior to one espresso (200 mg) this was counted as “yes”.

### Chronotype identification

The Composite Morningness Questionnaire is a self-assessment tool used to determine chronotype (Smith et al. 1989). It is comprised of 13 items, with responses to each item scored on a scale of 1 to 4 or 5, with a higher score representing a greater degree of morningness. Responses to all items are summed to create an overall score ranging from 13 to 55 with a higher score indicating an earlier chronotype. Chronotype scores were then divided into quintiles to determine chronotype groups (i.e., early, early intermediate, neutral, late intermediate, late). Participants were not aware of their chronotype whilst completing the study.

### Accelerometery

Participants were provided with an accelerometer (ActiGraph wGT3X-BT, ActiGraph, Pensacola, Florida, United States of America) to wear on their non-dominant wrist. They were instructed to wear the accelerometer continuously for the entire study duration (7 days), only removing the device to avoid contact with water (e.g., showering or swimming). Data sets with < 90% wear time were excluded from subsequent analyses. Data were collected at 30 Hz in 60 s epochs. The vector magnitude for each 60 s epoch was downloaded from the ActiLife software (Brønd et al. 2016). First, to help identify the time of peak activity during the day, and to help control for activity levels in subsequent linear models, a rolling window of 60 min was applied to the vector magnitude in order to calculate area under the curve (60-min AUC). Then, to identify the time of peak activity, the time of day of peak 60-min AUC for each 24-hour period was identified and averaged per participant across the 7 days. Finally, to obtain an arbitrary measure of overall activity for each participant, the total AUC over the 7 day period was calculated. Participants were asked to record any instances where they went swimming and therefore had to remove the accelerometer. Only one participant recorded two bouts of swimming, and was therefore removed. If participants chose to cycle during the study period, they were asked to move the accelerometer to their ankle and note the time of the activity in order not to lose activity time in the data set. Processed accelerometery levels were then examined and cycling vector magnitudes were found to be comparable to running magnitudes therefore were kept in the data set.

### Cognitive testing

Cognition was assessed via a version of the eXogenous-eNdogenous Attending (XNA) task adapted to be completed on mobile phones (Patel et al. 2025). The XNA task was designed to stress the cognitive control systems in the prefrontal cortex that enable the maintenance of attention to the external world (exogenous attending) in contrast to internalised thoughts (endogenous attending), and to voluntarily switch between one attending mode and the other (Burgess et al. 2007). The theoretical background and experimental tasks used to measure the operation of this system have been previously described (Benoit et al. 2012; Burgess and Wu 2013; Dumontheil et al. 2010). In addition to attentional processes, the XNA task combines elements of typical updating and working-memory type tasks, with the aim of stressing other executive control systems that have been demonstrated to be impacted by chronotype. This task was chosen as it is a useful representation of global executive functioning, and has been shown to have a high degree of construct validity (Burgess et al. 2022), therefore providing insights into cognitive control, attentional processes and working memory.

**Fig. 1:**
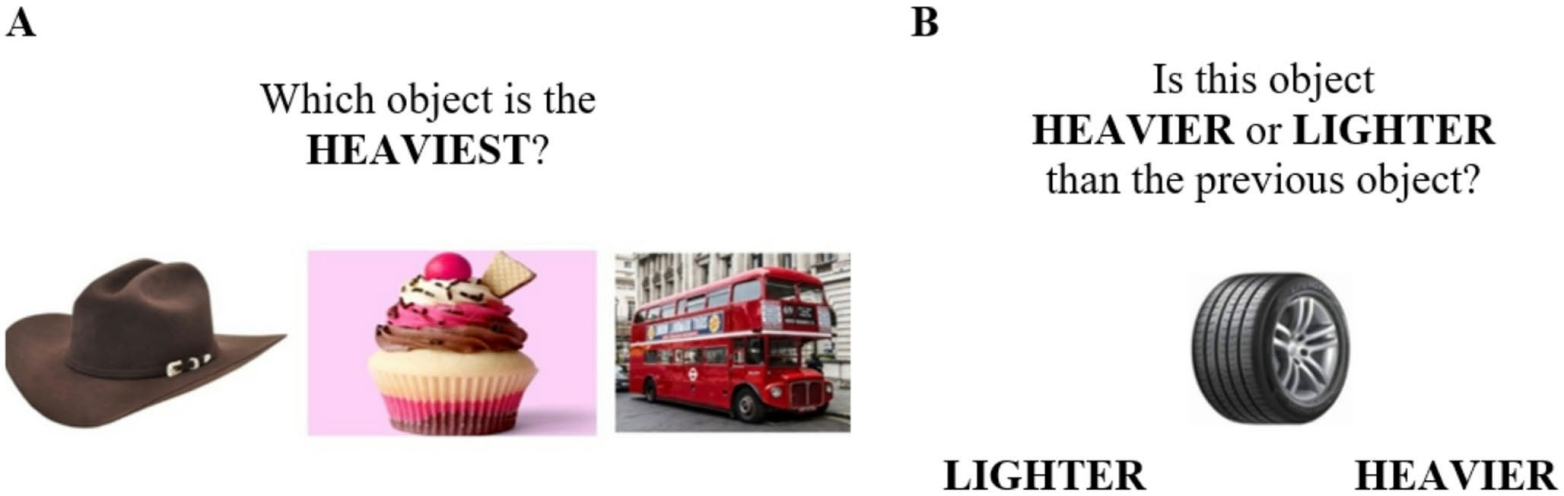
Example trials for XNA1 ** A** where participants were presented with images of three different objects and were asked to tap on the one they perceived to be the heaviest (17 total trials); and XNA2** B** where participants were presented with single objects in sequence, and were asked to indicate whether the current object was heavier or lighter than the previous one shown by tapping on the right or left of the mobile phone screen (27 total trials)

The first section of the task (XNA1, Fig. 1A) requires exogenous attending (also known as stimulus-oriented attending; Burgess et al. 2007) to compare weights of objects presented on the screen. The second part (XNA2, Fig. 1B) additionally requires endogenous attending, (or stimulus-independent attending) with an element of updating and working memory, to compare the weight of the presented object with one previously shown. In brief, XNA1 measures the cognitive control systems involved in attending to stimuli in the environment, whereas XNA2 measures the person’s ability to maintain thoughts in their head without being distracted (Burgess and Shallice 1996). XNA1 was always presented before XNA2. Six parallel versions of the tasks were created with different images to mitigate learning effects. Mean reaction times per task (XNA1 and XNA2) were calculated as the mean of all correct responses, after removing responses where reaction times were shorter than 125 ms or longer than 5000 ms (Whelan 2008). Errors were recorded as the number of trials where incorrect responses were made. Tests in which eight (for XNA1) or 10 (for XNA2) trials were completed incorrectly were removed from subsequent analysis on the assumption that the participant may not have been engaging appropriately with the task. On average, participants took five minutes to complete both XNA1 and XNA2.

### Measures of body composition

Body mass index (BMI) was calculated by dividing participant body mass in kilograms by the square of their height in metres. Height and weight were measured without shoes using a telescopic measuring rod fitted to scales (seca 704, seca, Germany). Waist to height ratio (WTHR) was calculated by dividing participant waist circumference by their height, both in centimetres. Waist circumference was measured with an anthropometric tape measure (NCD Medical Ltd., Ireland) taken at the umbilicus over one layer of clothing in a relaxed standing position. Body fat percentage was also estimated via bioelectrical impedance using a Tanita MC-980MA (Tanita Cooperation, Tokyo, Japan) device.

### Statistical analysis

Data analysis was completed in R Studio (Posit team 2025). Normality was checked using a Shapiro-Wilk test, and data was Box-Cox transformed where necessary (reaction time data for XNA1 and XNA2). Group mean outcomes were compared between chronotype groups using a one-way ANOVA with Bonferroni post-hoc tests. Linear mixed models with repeated measures (random effects per participant) were used to investigate associations between cognitive performance and chronotype, including an interaction for time of day. Chronotype was added as a continuous variable, the overall questionnaire score. Time of day of cognitive task completion was also continuous as this varied between participants and days due to the free-living nature of the study. Age, sex, caffeine intake one hour prior to completing the test and physical activity level within one hour prior to the cognitive test (as determined via 60 min AUC) were controlled for. A significance level of α = 0.05 is used for all analysis.

## Results

Seventy-six participants took part in the study. Of these, one was excluded due to incomplete cognitive data and poor accelerometer wear time. Therefore, seventy-five young adults (25 ± 7 years old, 57% female) completed the study. These included university students (*n* = 59), early career researchers (*n* = 7), and members of the public (*n* = 9), all of whom had full control of their working hours. There were no significant differences in sex, age, BMI, WTHR or body fat percentage between chronotype groups (Table 1). Although participants were prompted to provide responses at set times (09:00 and 21:00), these were variable (Fig. S1).

**Table 1:** Demographic characteristics of the total study sample (*n* = 75) and split by chronotype group

Chronotype	*N* participants (female)	University students	Age (mean ± SD)	BMI (mean ± SD)	WTHR (mean ± SD)	Body fat % (mean ± SD)
Early	15 (10)	11	26 ± 7	22.73 ± 2.91	0.46 ± 0.05	20.24 ± 4.90
Early Intermediate	15 (9)	11	25 ± 7	23.75 ± 3.27	0.48 ± 0.05	23.2 ± 6.19
Neutral	13 (6)	11	23 ± 7	21.49 ± 2.69	0.45 ± 0.04	18.57 ± 6.23
Late Intermediate	16 (10)	14	23 ± 5	23.39 ± 2.45	0.46 ± 0.04	22.03 ± 8.16
Late	16 (8)	12	26 ± 8	22.89 ± 2.51	0.48 ± 0.07	21.89 ± 8.77
All	75 (43)	59	25 ± 7	22.92 ± 2.80	0.47 ± 0.05	21.33 ± 7.08

### Activity levels by chronotype group

**Fig. 2:**
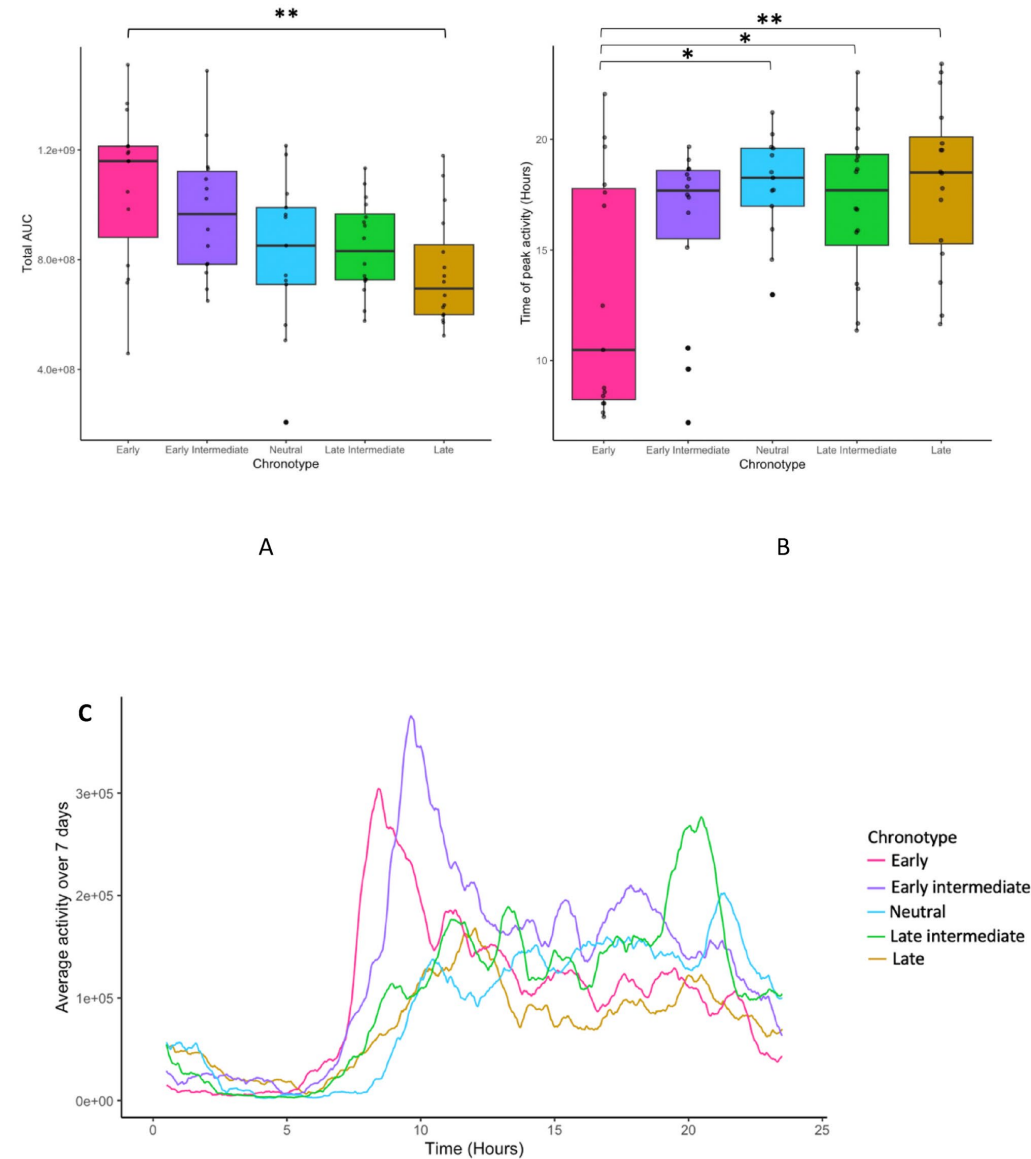
**A** Total activity levels by chronotype group, using 7 day AUC per participant across the study period. **B** Mean time of peak daily activity by chronotype group. The early chronotype group were active significantly earlier than the neutral, late-intermediate and late chronotype groups. * *p* < .05, ** *p* < .01. **C** The average 60-min AUC rolling window, averaged first within person over a 24 hour period for the full 7 days, and then averaged within chronotype group

There was a main effect of chronotype for physical activity behaviours (*Fs*(4,69) = 4.27 and 4.24, ps = 0.004, η^2^*p* = .20). Early chronotypes were more active (7 day AUC) compared to late chronotypes (*p* = .004) (Fig. 2A) and they had an earlier time of peak activity compared to neutrals (*p* = .01), late intermediates (*p* = .03), and late chronotypes (*p* = .005), respectively (Fig. 2B and C).

### Cognitive data

Within-subject multiple linear models were conducted on cognitive task perfomance to investigate associations with chronotype and time of day after controlling for key covariates (age, sex, caffeine and physical activity in the previous hour; Table 2). Main effects of chronotype revealed that a higher score on the morningness scale (earlier chronotype) was associated with shorter reaction times on XNA1 (t(109) = −3.01, *p* = .003) and XNA2 (t(111) = −2.88, *p* = .005) and fewer errors on XNA1 (t(309) = −3.15, *p* = .008). Main effects of time of day showed significant association between a later time of day and better cognitive performance as measured by the same variables ( ps < 0.001). Thus, both earlier chronotype and later time of day were independently associated with better cognitive performance. Raw cognitive performance variables, by chronotype group and time of day, are available in the supplementary material (Table S1).

**Fig. 3:**
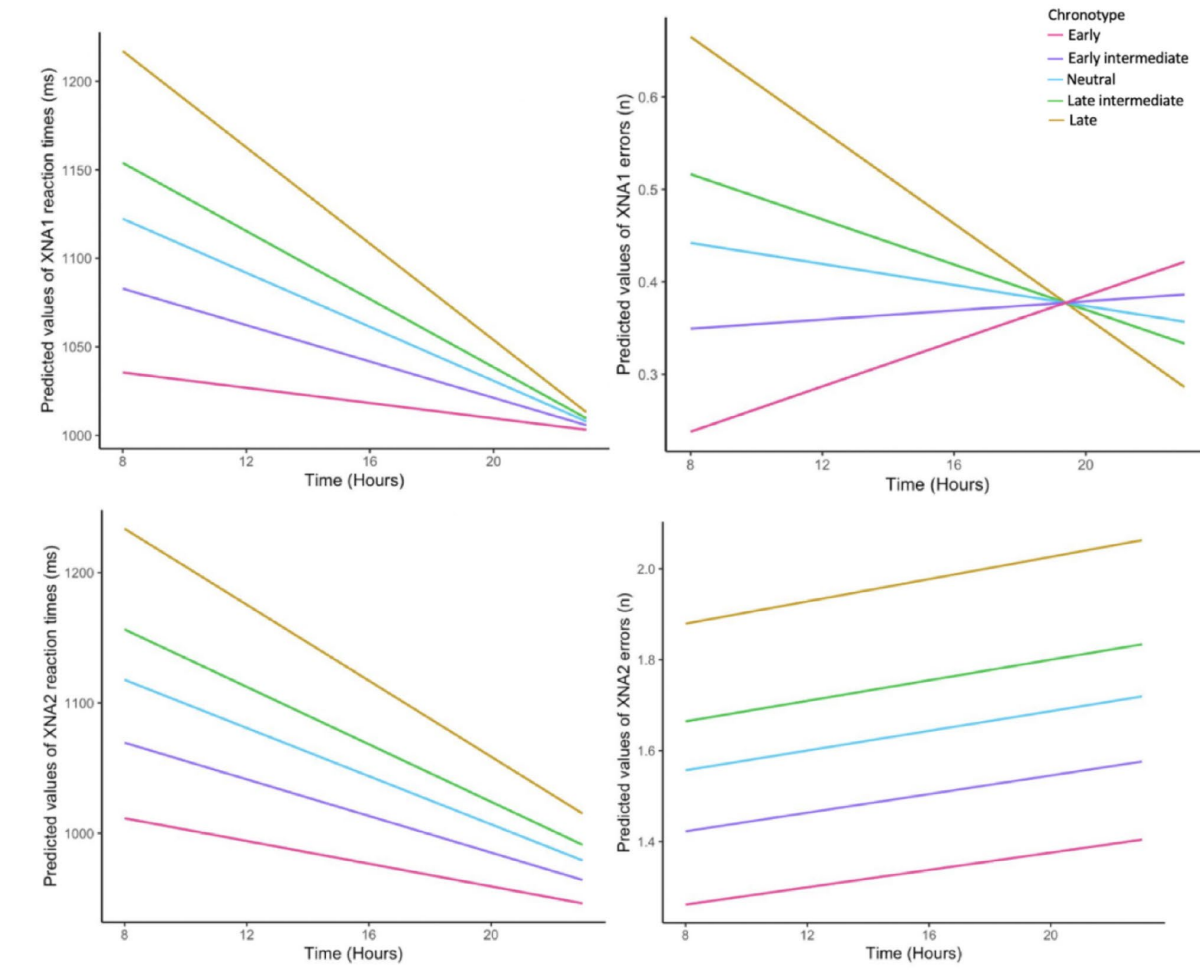
Change in cognitive performance, as measured using XNA reaction times (ms) and errors (n trials), across a 24 hour period, by chronotype group, after controlling for age, sex, caffeine and physical activity in the past hour. Full results are shown in Table 2

Furthermore, there were significant interactions between chronotype and time, where a later chronotype was associated with greater change in cognitive performance across the day (*p* < .005). That is, results suggest that early chronotypes performed better in the morning and showed more consistent cognitive performance throughout the day compared to late chronotypes. The latter performed more poorly in the morning but showed greater improvement towards later hours of the day (Fig. 3). This effect was evident for XNA1 reaction times (t(463) = 3.82, *p* < .001) and errors (t(492) = 3.26, *p* = .001), and for XNA2 reaction times (t(465) = 2.79, *p* = .005) but not errors. Of note, higher physical activity levels in the hour prior to task completion was associated with fewer XNA1 errors (t(293) = −2.03, *p* = .04) but no other measure of cognitive performance.

**Table 2:** Linear mixed model outputs investigating the effect of time of day and chronotype on XNA reaction times (ms) and errors (n trials)

Predictors	Estimate	95% CI (LL – UL)	*p*
*XNA1 Reaction Time*			
(Intercept)	1195.21	978.15c1412.26	< 0.001
Time of day	−15.65	−20.02 – −11.29	< 0.001
Chronotype score	−13.39	−21.67 – −5.11	0.002
Chronotype * Time of day	0.54	0.27–0.81	< 0.001
Age	7.39	−0.42–14.36	0.038
Sex (Male)	12.15	−87.51–111.82	0.811
Caffeine in the past hour (Yes)	29.68	−25.71–85.07	0.293
Activity in previous 60 min	−3e-05	−2e-04 − 2e-04	0.765
*XNA1 Errors*			
(Intercept)	0.92	0.45–1.38	< 0.001
Time of day	−0.03	−0.05 – −0.01	< 0.001
Chronotype score	−0.03	−0.05 – −0.01	0.002
Chronotype * Time of day	1.6e-03	1.3e-03– 2.6e-03	0.001
Age	0.005	−0.01–0.02	0.458
Sex (Male)	−0.13	−0.30–0.05	0.165
Caffeine in the past hour (Yes)	−0.10	−0.30–0.10	0.315
Activity in previous 60 min	−9e-07	−2e-06 – −3e-08	0.042
*XNA2 Reaction Time*			
(Intercept)	1211.74	959.65–1463.84	< 0.001
Time of day	−16.44	−21.78 – −11.09	< 0.001
Chronotype score	−14.81	−24.50 – −5.13	0.003
Chronotype * Time of day	0.48	0.15–0.82	0.005
Age	7.42	−0.60–15.44	0.070
Sex (Male)	80.43	−34.54–195.41	0.170
Caffeine in the past hour (Yes)	4.92	−62.43–72.26	0.886
Activity in previous 60 min	2e-04	−7e-04–1e-03	0.195
*XNA2 Errors*			
(Intercept)	3.99	1.39–6.58	0.003
Time of day	0.01	−0.06–0.09	0.689
Chronotype score	−0.03	−0.09–0.04	0.427
Chronotype * Time of day	−1.1e-04	−0.001–0.001	0.913
Age	−0.06	−0.12 – −0.01	0.029
Sex (Male)	−0.13	−0.95–0.68	0.751
Caffeine in the past hour (Yes)	0.10	−0.35–0.56	0.662
Activity in previous 60 min	−3e-09	−2e-06–2e-06	0.997

Random effects per participant to account for repeated measures are included in each model.

## Discussion

In this naturalistic observation on the associations between chronotype, physical activity, and cognitive performance, participants with earlier chronotypes were more physically active and demonstrated peak activity times earlier in the day. A higher degree of morningness was also associated with better performance on tasks measuring exogenous and endogenous attending, particularly in the morning. Although later chronotypes performed worse than earlier chronotypes in the morning (with reaction time differences of ~ 200 ms), the former exhibited an improvement in cognitive performance towards the evening, where group differences became minimal. Of note, physical activity prior to completing the cognitive task predicted fewer errors in exogenous attending, but not other metrics.

The higher, and earlier, physical activity levels observed in the earlier chronotypes align with recent literature. Indeed, individuals with early chronotypes have been shown to be more physically active (Nauha et al. 2020; Polańska et al. 2024), and to be active earlier in the day (Glavin et al. 2020; Hicks et al. 2023). It is important to recognise, however, that the work by Hicks et al. (2023) did not find significant differences in total physical activity between chronotype groups, potentially due to their sample consisting of older adults. These results are in keeping with a review by Sempere-Rubio and colleagues who concluded that the relationship between chronotype and physical activity requires more research across different ages, including in a young, university-aged population (Sempere-Rubio et al. 2022). Other studies have aimed to develop a mechanistic understanding of these purported effects. For example, a genome-wide association study completed by Klimentidis et al. (2018) which used the UK Biobank, demonstrated genetic correlations between habitual physical activity uptake and early chronotypes. The authors indicated a potential genetic propensity for these individuals to exercise more frequently (Klimentidis et al. 2018). In turn, physical activity has also been shown to act as a synchronizer for the circadian system, potentially via its effects on melatonin and in turn the interaction between sleep, melatonin and activity (Montaruli et al. 2017). However, the ability of physical activity to entrain circadian rhythm must be interpreted in the context of the known genetic basis of chronotype (Kalmbach et al. 2017).

Given the known beneficial effects of physical activity on cognitive performance (Crum et al. 2024; Zhang et al. 2023), these differences in behaviour were accounted for when examining the associations between chronotype and attendance. In fact, mechanistic studies have suggested that task performance may relate to changes in biomolecule concentrations which facilitate cognitive function at a given time of day, and which might be further modulated by physical activity. For example, norepinephrine and acetylcholine concentrations have been shown to closely follow circadian rhythms, where the latter is closely related to physiological arousal levels and involved in processes associated with sleep (Baghdoyan et al. 1993; Kalanadhabhatta et al. 2021; Matchock and Mordkoff 2008). Schmidt and colleagues (2015) have in fact observed differences in frontal lobe and thalamic activity between chronotypes during a 3-back task, which aligned with their circadian preference. Taken together, it may be that chronotype is a manifestation of biological and neurological processes which fluctuate over the course of the day and may support cognitive function.

The improvement in cognitive performance observed in late chronotypes throughout the day partially confirms the synchrony effects demonstrated by a few prior studies with regard to late chronotypes, but not for early ones. This literature has provided evidence that an individual’s chronotype aligns with the time of day when their cognitive performance is optimal, including when using semantic analogy (Nowack and Van Der Meer 2018) and fluid intelligence (Goldstein et al. 2007) tasks. Goldstein et al. (2007) highlighted that, beyond the synchrony effect, late chronotype adolescents also exhibited maladaptive behaviours, putting them at greater risk of challenges with academic engagement. The results presented here somewhat support Goldstein et al.’s (2007) conclusions, as young adults with later chronotypes had worse performance on tasks measuring attentional processes in the morning compared to early chronotypes. The latter, however, performed consistently better on the cognitive battery throughout the day. While this study did not measure intelligence, the XNA task implemented here provides insight into cognitive control and attentional processes required to attend to the environment, to internalise thoughts without distraction and to engage elements of working memory that are necessary for executive functioning. Notably, the XNA task itself has also demonstrated a high degree of construct validity (Burgess et al. 2022), making it a useful representation of daily functioning. However, these results differ from prior findings suggesting better cognitive performance of evening types, irrespective of time of day (Ceglarek et al. 2021; West et al. 2024). This evidences the highly complex relationship between chronotype, time of day and cognitive performance (Munnilari et al. 2024). For example, the synchrony effect has not been consistently shown across cognitive domains, with some even benefitting at ‘non-optimal’ times of day (Adan et al. 2012).

Regarding the effects of physical activity in this context, the negative association between late chronotype and task performance was positively influenced by activity behaviours. The positive main effect of physical activity on task accuracy, regardless of chronotype effects, provides a potential promising avenue for the development of lifestyle interventions to support morning cognitive performance in those most impacted by such temporal effects.

### Limitations

Due to the observational nature of the study, several limitations are noted. First, although participants were asked to complete the questionnaires and cognitive tests at specific times (i.e., 09:00 and 21:00 h), actual completion times were highly variable. Although enabling a more naturalistic design, this variability allows for the possibility that participants completed testing at their preferred times, thereby potentially skewing results. Indeed, individuals with later chronotypes often completed their first tests after 09:00 h. Second, physical activity was quantified using accelerometers, which is a robust objective measure of movement and more reliable than self-reporting. However, the type of exercise undertaken by participants was not recorded. This is a salient factor given that exercise intensity, duration and modality have been shown to moderate the degree to which exercise affects cognition (Chang et al. 2012). Thirdly, cognitive testing was completed by participants at home using mobile phones. Although this testing method may be a better representation of participants’ cognitive performance in a real-world environment, it is likely to introduced greater variability than that expected in more controlled, lab-based conditions. Last, differences in lifestyle behaviours such as sleep timing, day-time commitments, social jetlag, intra-week fluctuations, differences between weekdays and weekends are all factors that are likely to shape daily living patterns as well as cognitive performance and may have influenced the results. Future work could consider the effects of these elements on daily activity patterns in different chronotypes to aid the development of inclusive scheduling.

## Conclusion

These results provide additional evidence that chronotype is associated with both cognitive performance and physical activity over the course of a day, with promising naturalistic evidence that the latter might support cognition regardless of chronotype. This work might be used as a first step in better understanding ways in which physical activity may be best leveraged to support cognitive performance in real-world contexts, particularly for individuals most impacted by temporal effects of their chronotype.

## Supplementary Information

Below is the link to the electronic supplementary material.

Supplementary Material 1

## Data Availability

Data are available from the corresponding author upon reasonable request.
