# Supplementary material for: Associations between chronotype, physical activity and cognition in a free-living setting
Source: Exp Brain Res. 2026 Feb 27;244(4):53. doi: 10.1007/s00221-026-07254-5 (PMC12948795; doi:10.1007/s00221-026-07254-5)
Supplement: Supplementary file 1 — Supplementary Material 1 [file 221_2026_7254_MOESM1_ESM.docx]

**Supplementary materials**


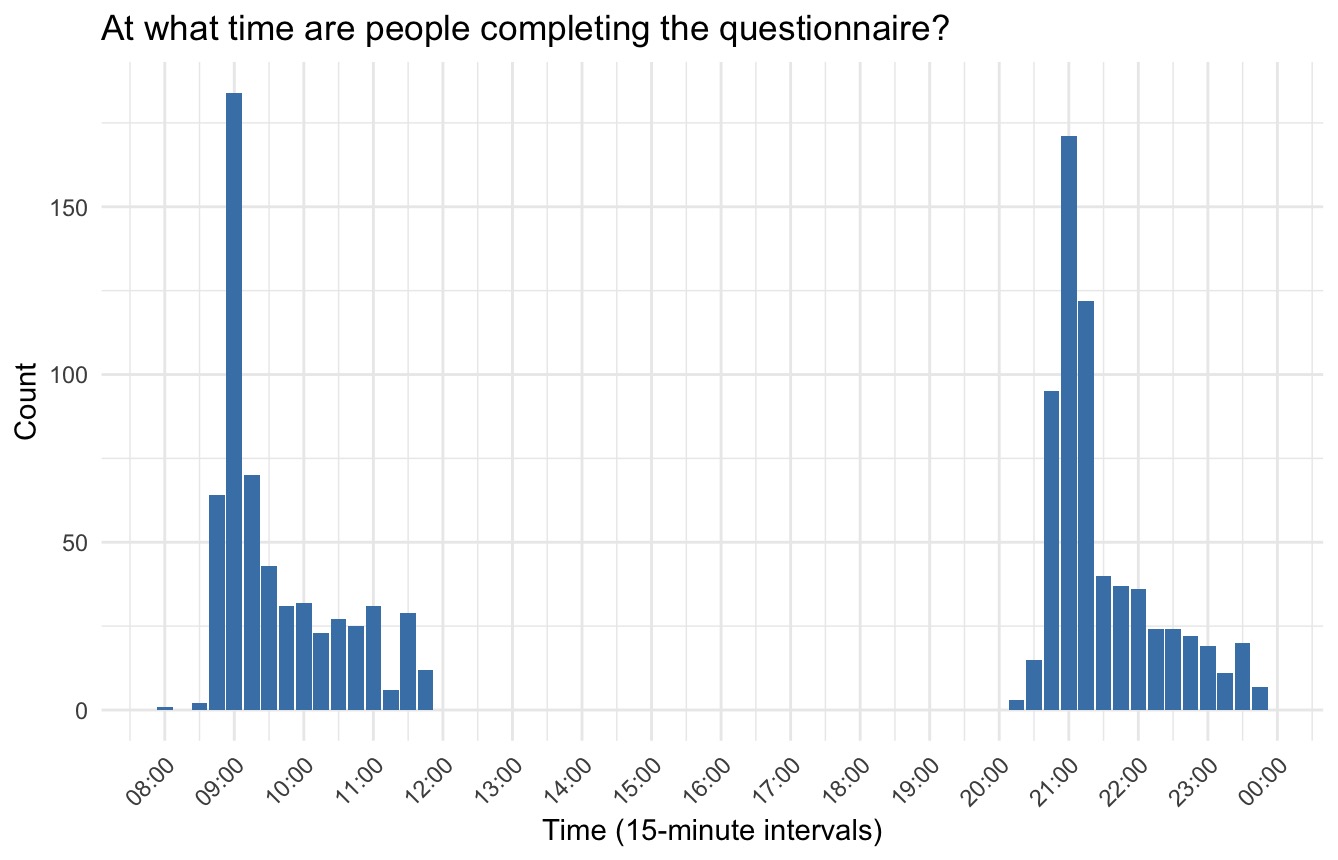


Number of cognitive tasks completed

Figure S1. Number of cognitive tasks completed, by time of day, across the study period.

| **Table S1.** Performance on cognitive tasks, by chronotype group and time of cognitive testing (morning and evening, at approximately 09:00 and 21:00 respectively). Cognitive performance is measured using reaction time (RT, ms) and errors (n of trials) on parts 1 and 2 of the XNA task. | | | | | | | | |
| --- | --- | --- | --- | --- | --- | --- | --- | --- |
| **Chronotype group** | **XNA 1 RT** | | **XNA 2 RT** | | **XNA 1 errors** | | **XNA 2 errors** | |
|  | **Morning** | **Evening** | **Morning** | **Evening** | **Morning** | **Evening** | **Morning** | **Evening** |
| Early | 1050.49 ± 290.50 | 1030.99 ± 210.84 | 1070.31 ± 290.33 | 1040.61 ± 247.43 | 0.27 ± 0.49 | 0.46 ± 0.79 | 1.42 ± 1.24 | 1.23 ± 1.19 |
| Early Intermediate | 1191.36 ± 228.69 | 1093.42 ± 185.60 | 1172.53 ± 235.18 | 1061.30 ± 207.89 | 0.29 ± 0.56 | 0.11 ± 0.38 | 1.98 ± 2.34 | 2.66 ± 2.64 |
| Neutral | 1220.95 ± 312.45 | 1116.60 ± 293.15 | 1262.00 ± 292.91 | 1144.37 ± 275.01 | 0.22 ± 0.46 | 0.16 ± 0.37 | 0.90 ± 1.08 | 1.22 ± 1.43 |
| Late Intermediate | 1143.87 ± 228.91 | 975.54 ± 166.73 | 1195.16 ± 322.27 | 1089.33 ± 199.16 | 0.33 ± 0.48 | 0.66 ± 0.55 | 1.57 ± 1.64 | 1.35 ± 1.48 |
| Late | 1308.39 ± 399.78 | 1126.71 ± 245.13 | 1309.10 ± 410.25 | 1121.16 ± 266.19 | 0.77 ± 0.96 | 0.33 ± 0.66 | 2.60 ± 2.27 | 2.98 ± 2.72 |
